# Malaria among under-five children in Ethiopia: a systematic review and meta-analysis

**DOI:** 10.1186/s12936-022-04370-9

**Published:** 2022-11-16

**Authors:** Gebeyaw Biset, Abay Woday Tadess, Kirubel Dagnaw Tegegne, Lehulu Tilahun, Natnael Atnafu

**Affiliations:** 1 Department of Pediatrics and Child Health Nursing, School of Nursing and Midwifery, College of Medicine and Health Sciences, Wollo University, Dessie, Ethiopia; 2 Dream Science and Technology College, Dessie, Ethiopia; 3 Department of Public Health, College of Medicine and Health Sciences, Samara University, Samara, Ethiopia; 4 Department of Adult Health Nursing, School of Nursing and Midwifery, College of Medicine and Health Sciences, Wollo University, Dessie, Ethiopia; 5 Department of Emergency and Critical Care Nursing, School of Nursing and Midwifery, College of Medicine and Health Sciences, Wollo University, Dessie, Ethiopia; 6 School of Midwifery, College of Health Science and Medicine, Wolaita Sodo University, Wolaita Sodo, Ethiopia

**Keywords:** Under-five children, Malaria, Systematic review, Meta-analysis, Ethiopia

## Abstract

**Background:**

Globally, malaria is among the leading cause of under-five mortality and morbidity. Despite various malaria elimination strategies being implemented in the last decades, malaria remains a major public health concern, particularly in tropical and sub-tropical regions. Furthermore, there have been limited and inconclusive studies in Ethiopia to generate information for action towards malaria in under-five children. Additionally, there is a considerable disparity between the results of the existing studies. Therefore, the pooled estimate from this study will provide a more conclusive result to take evidence-based interventional measures against under-five malaria.

**Methods:**

The protocol of this review is registered at PROSPERO with registration number CRD42020157886. All appropriate databases and grey literature were searched to find relevant articles. Studies reporting the prevalence or risk factors of malaria among under-five children were included. The quality of each study was assessed using the Newcastle–Ottawa Quality Assessment Scale (NOS). Data was extracted using Microsoft Excel 2016 and analysis was done using STATA 16.0 statistical software. The pooled prevalence and its associated factors of malaria were determined using a random effect model. Heterogeneity between studies was assessed using the Cochrane Q-test statistics and I^2^ test. Furthermore, publication bias was checked by the visual inspection of the funnel plot and using Egger’s and Begg’s statistical tests.

****Result**s:**

Twelve studies with 34,842 under-five children were included. The pooled prevalence of under-five malaria was 22.03% (95% CI 12.25%, 31.80%). Lack of insecticide-treated mosquito net utilization (AOR: 5.67, 95% CI 3.6, 7.74), poor knowledge of child caretakers towards malaria transmission (AOR: 2.79, 95% CI 1.70, 3.89), and living near mosquito breeding sites (AOR: 5.05, 95% CI 2.92, 7.19) were risk factors of under-five malaria.

**Conclusion:**

More than one in five children aged under five years were infected with malaria. This suggests the rate of under-five malaria is far off the 2030 national malaria elimination programme of Ethiopia. The Government should strengthen malaria control strategies such as disseminating insecticide-treated mosquito nets (ITNs), advocating the utilization of ITNs, and raising community awareness regarding malaria transmission.

**Supplementary Information:**

The online version contains supplementary material available at 10.1186/s12936-022-04370-9.

## Background

Malaria is a major public health concern in tropical and sub-tropical regions of the world, affecting hundreds of millions of people. Nearly 3.2 billion people are at risk of malaria worldwide with a substantial risk among pregnant women and children under five years old. In the year 2020, nearly 241 million people were infected by *Plasmodium* species and half a million died due to malaria. Evidence suggested that more than 95% of malaria infections and deaths are concentrated in African countries. Similarly, more than 90% of malaria-related infections and deaths occur in sub-Saharan regions [1, 2].

Malaria is a major public health problem in Ethiopia where approximately 68% of the land mass has favourable conditions for malaria transmission and 60% of the population is at risk of the disease [3, 4]. Despite various preventive measures undertaken in the last decades, malaria remains among the top ten causes of under-five morbidity and mortality in Ethiopia [5–8]. Malaria transmission is highly seasonal and varies significantly with respect to geographical altitude, rainfall and population movement. In addition, due to unstable malaria transmission patterns, Ethiopia is prone to focused and large-scale cyclic malaria epidemics [9].

Studies show that children aged under five years are among the most vulnerable to malaria infections. More than 61% of all malaria deaths worldwide and 80% of sub-Saharan malaria deaths occurred among children under 5 years [10, 11]. Similarly, the highest malaria-related morbidity and mortality in Ethiopia is reported among children under 5 years [12–15]. The 2019 Ethiopian demographic health survey (EDHS) showed that 59 under-fives died in 1,000 live births due to malaria [16].

Despite inconsistency among existing studies, several factors were associated with high prevalence of malaria among children under 5 years. Insecticide-treated mosquito net (ITN) utilization, presence of forest cover, altitude of residence, household density, living near mosquito breeding sites such as stagnant water, seasonal variation, and housing conditions were major predictors of malaria infection among children aged under 5 years. In addition, the low protective immunity of under-fives make them highly susceptible to malaria infection [17–19].

Several malaria elimination strategies have been implemented at national and international levels to control the burden of malaria. Consequently, over 6 million malaria deaths were averted between 2000 and 2015 in African countries. Despite this significant decline, malaria remains a major public health concern in tropical and sub-tropical regions of the world [20–22]. Furthermore, there are limited and inconclusive studies in Ethiopia to generate information for action against malaria. There is a considerable discrepancy among the results of existing studies. Therefore, the pooled estimate from the current study will provide a more conclusive result to take evidence-based interventional measures against malaria in under-fives in Ethiopia [23].

## Methods

### Study design

This study was designed based on the updated guideline of the Preferred Reporting Items for Systematic Review and Meta-analyses (PRISMA 2020) statements to report the findings [24]. The protocol has been registered at PROSPERO with registration number CRD42020157886. Moreover, the authors have used the guideline of PROSPERO for registering the protocol.

### Eligibility criteria

The inclusion criteria for this review and meta-analysis were: (1) studies among under-five children in Ethiopia; (2) observational studies (cross-sectional, case-control, cohort studies, longitudinal studies); (3) studies that report the prevalence or factors of under-five malaria; and, (4) English language articles published in peer-reviewed journals not limited by year of study. However, studies that did not define the age of a child, studies that were not fully accessed or failed to contact the primary author (s), case reports, expert opinions, and qualitative studies were excluded.

### Searching strategy

This review and meta-analysis was prepared and presented in accordance to the updated guideline of the Preferred Reporting Items for Systematic Reviews and Meta-Analysis (PRISMA 2020) [24]. The international databases PubMed/MEDLINE, HINARI, African Journal of Online (AJOL), and Google Scholar were accessed to find relevant articles. A Medical Subject Headings (MeSH), keyword terms and phrases were used both in separation and in combination using the Boolean operators “OR” and “AND” to search for eligible articles. The authors have also used snowballing of the references of identified articles for accessing potentially relevant studies (Additional file 1).

The keywords and phrases “under-five children”, “0–59 months old children”, pediatrics, ‘‘preschool children’’, malaria, “*Plasmodium falciparum*”, “*Plasmodium vivax*”, “mixed malaria”, “*Plasmodium* species”, prevalence, incidence, magnitude, burden, proportion, determinants, “risk factors”, predictors, causes, ‘‘associated factors”, and Ethiopia were used in separation or in combination to retrieve relevant articles on malaria in under-fives in Ethiopia.

### Study selection

**Fig. 1:**
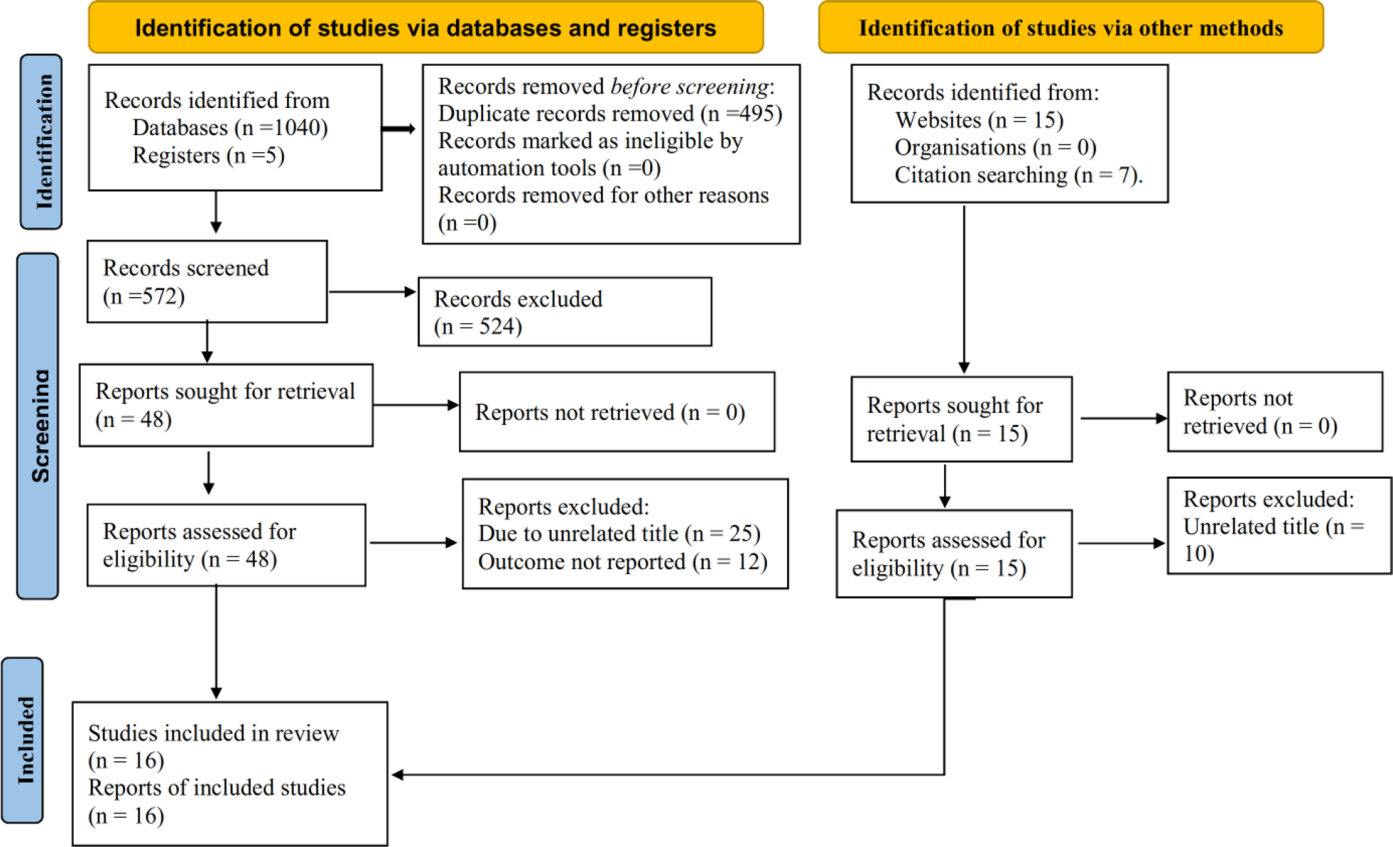
Flow chart illustrating the process of literature search and selection of studies included in the present systematic review and meta-analysis

Exhaustively, 1067 studies were retrieved using online databases, reference lists of retrieved articles, and manual searches. All the retrieved articles were exported to Endnote X8 reference managers and 495 articles were removed due to duplication. The title and abstract of the remaining 572 articles were reviewed and 524 articles were removed by title and abstract. Some 48 full articles were assessed for eligibility, which resulted in further exclusion of 36 articles due to lack of outcome reports. Finally, 16 studies were included. Of these 16, 12 studies were used to determine the pooled prevalence of malaria, 11 studies to determine the association between mosquito net utilization and under-five malaria, 5 studies to determine the association between living near mosquito breeding sites and under-five malaria, and 5 studies to determine the association between knowledge of malaria transmission and the incidence of under-five malaria (Fig. 1).

### Outcome measurement

The primary outcome of this systematic review and meta-analysis is the pooled prevalence of malaria among under-five children. The pooled prevalence of malaria was calculated by dividing the number of under-fives with malaria by the total number of children included in the study multiplied by 100. The second outcome was determinants of malaria among under-five children, which was estimated by the pooled odds ratio with a 95% confidence interval using a random effect meta-analysis.

### Data extraction and management

Data were extracted using Microsoft Excel spreadsheet imported into STATA version 16.0 statistical software for further analysis. Three authors (AW, KD, GB) extracted the data independently. Discrepancies between authors during data extraction were managed through consensus and a fourth author was consulted to resolve disagreements. For each included article, the first author’s last name, year of publication, study setting, study design, study period, sample size, response rate, study population, outcome definition, comparison groups, and effect estimate was recorded.

### Quality assessment

The quality of each study was assessed using NOS adapted for meta-analysis. Studies were assessed for representativeness of sample size, non-respondents, ascertainment of exposure, comparability, assessment of the outcome, and statistical tests. Stars were assigned to evaluate study quality, with 9–10 stars indicating “very good” quality, 7–8 stars “good” quality, 5–6 stars “satisfactory” quality, and 0–4 stars “unsatisfactory” quality. Four authors (GB, AW, KD, NA) performed the quality appraisal independently and the average assessment scale was used for the final decision [25].

### Publication bias and heterogeneity

Heterogeneity was evaluated using Cochran’s Q statistic with a 5% level of significance and the I^2^ statistical test. The I^2^ test statistics of 25, 50 and 75% were declared as low, moderate, and high heterogeneity respectively [26]. The possible risk of publication bias was examined by the inspection of the funnel plot and statistically using Begg’s correlation and Egger’s regression test. Sub-group analysis was conducted by region, sample size and study design to minimize random variation among studies. Besides, sensitivity analysis was performed to examine the influence of a single study on the overall estimate.

### Data analysis

The prevalence of malaria was calculated by dividing the number of under-five children with malaria by the total number of children included in the study multiplied by 100. The standard error of the prevalence was calculated using the formula: $se\;\left(p\right)=\frac{\sqrt{\text{p}(1-\text{p})}}{n}$. Adjusted odds ratio (AOR) with its upper and lower bounds were extracted for significant variables. The standard error of the OR was calculated using the formula: $\text{SE}\; \log \;(\text{OR})=\left[\frac{UB\;cl-LB\;cl}{2Za/2}\right]$. Then the extracted data were exported into STATA 16.0 statistical software for further analysis. Taking the variability between individual studies and the observed heterogeneity among the included studies, a random effect model analysis was employed. Additionally, sub-group analysis, publications bias and sensitivity analysis were performed.

## Results

### Study characteristics

Of the 16 included studies, five were in Amhara region, three in Oromia region, four in South Nation Nationality and Peoples of Ethiopia (SNNP) region, two in Binishangulgumz, one in Afar region, and one was a national study. The highest malaria prevalence was reported in Afar region (64.04%) [27], whereas the lowest prevalence was reported in Binishangulgumz (3.93%) [28]. Regarding the study design, 12 studies were cross-sectional, three retrospectives, and one Bayesian study (Table 1).

**Table 1:** Characteristics of studies included in the systematic review and meta-analysis of prevalence and/or associated factors of malaria in Ethiopia, August 2022

Author [Pub year]	Region	Study design	Sample size	Prevalence	NOS score
Abrham et al. [2017]	Amhara	Cross sectional	419	9.1	7 (low risk)
Abossie et al. [2020]	SNNP	Cross sectional	271	22.14	6 (low risk)
Ahmed et al. [2021]	Binishangul	Cross sectional	356	3.93	7 (low risk)
Alkadir et al. [2020]	Binishangul	Retrospective	8660	15.88	8 (low risk)
IFA AC et al. [2018]	SNNP	Retrospective	5210	47.2	7.5 (low risk)
Legesse et al. [2019]	SNNP	Cross sectional	271	24.35	6.5 (low risk)
Shiferaw et al. [2018]	Amhara	Retrospective	12,096	20.54	7.5 (low risk)
Woday et al. [2019]	Afar	Cross sectional	484	64.04	7.5 (low risk)
Deressa et al. [2007]	Oromia	Cross sectional	3873	21.1	7 (low risk)
Deribew et al. [2010]	SNNP	Cross sectional	2410	9.42	7 (low risk)
Tsegaye et al. [2017]	Amhara	Cross sectional	585	8.72	8 (low risk)
Workineh et al. [2021]	Amhara	Cross sectional	207	18.36	7.5 (low risk)
Graves et al. [2009]	Oromia	cross sectional	11,538	–	7 (low risk)
Hailu et al. [2018]	Amhara	Cross sectional	333	–	7.5 (low risk)
Aychiluhm et al. [2020]	Ethiopia	Bayesian	8301	–	8 (low risk)
Abdishu et al. [2022]	Oromia	cross sectional	504	–	7.5 (low risk)

### Prevalence of malaria in under-five children

**Fig. 2:**
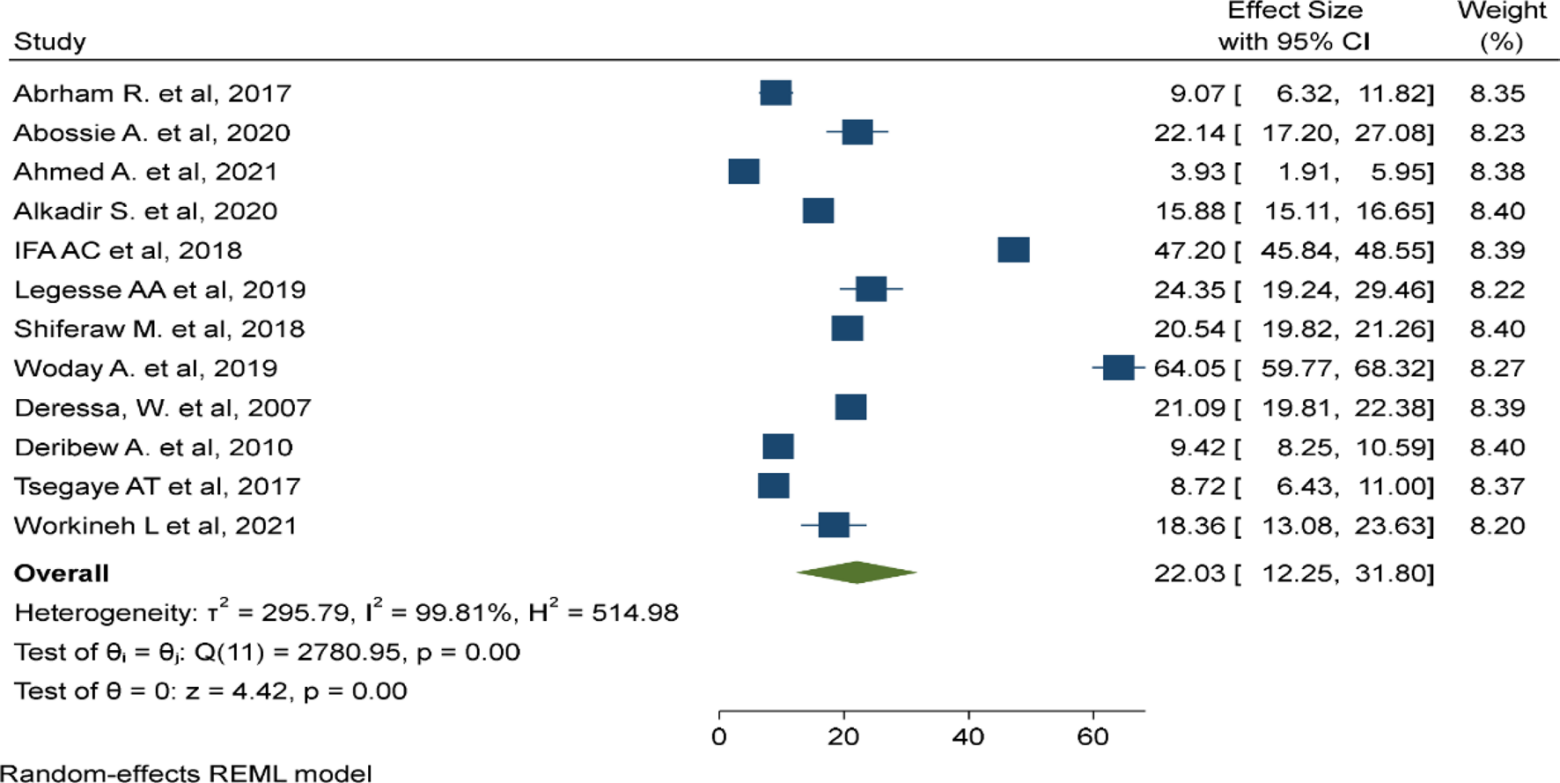
Pooled prevalence malaria among under-five children in Ethiopia, August 2022

In this meta-analysis, 12 primary studies with 34,842 study participants were included to estimate the pooled prevalence of malaria among under-five children using a random effect model [27–38]. The pooled prevalence of malaria among under-five children was 22.03% (95% CI 12.25%, 31.8%, I^2^: 99.81, P < 0.001) (Fig. 2).

### Sub-group analysis

**Fig. 3:**
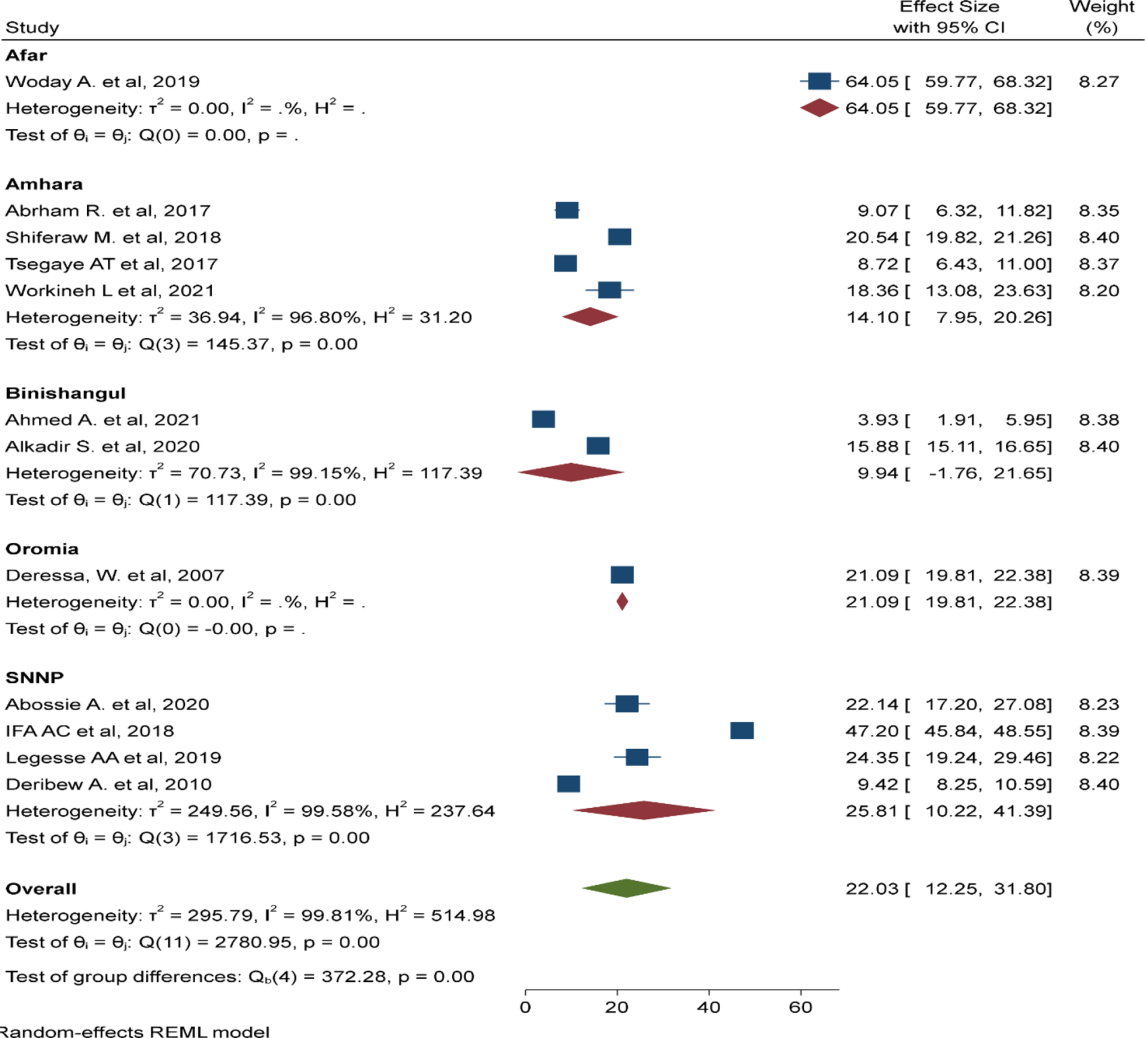
Subgroup analysis based on the region where the studies are conducted, Ethiopia, August 2022

**Fig. 4:**
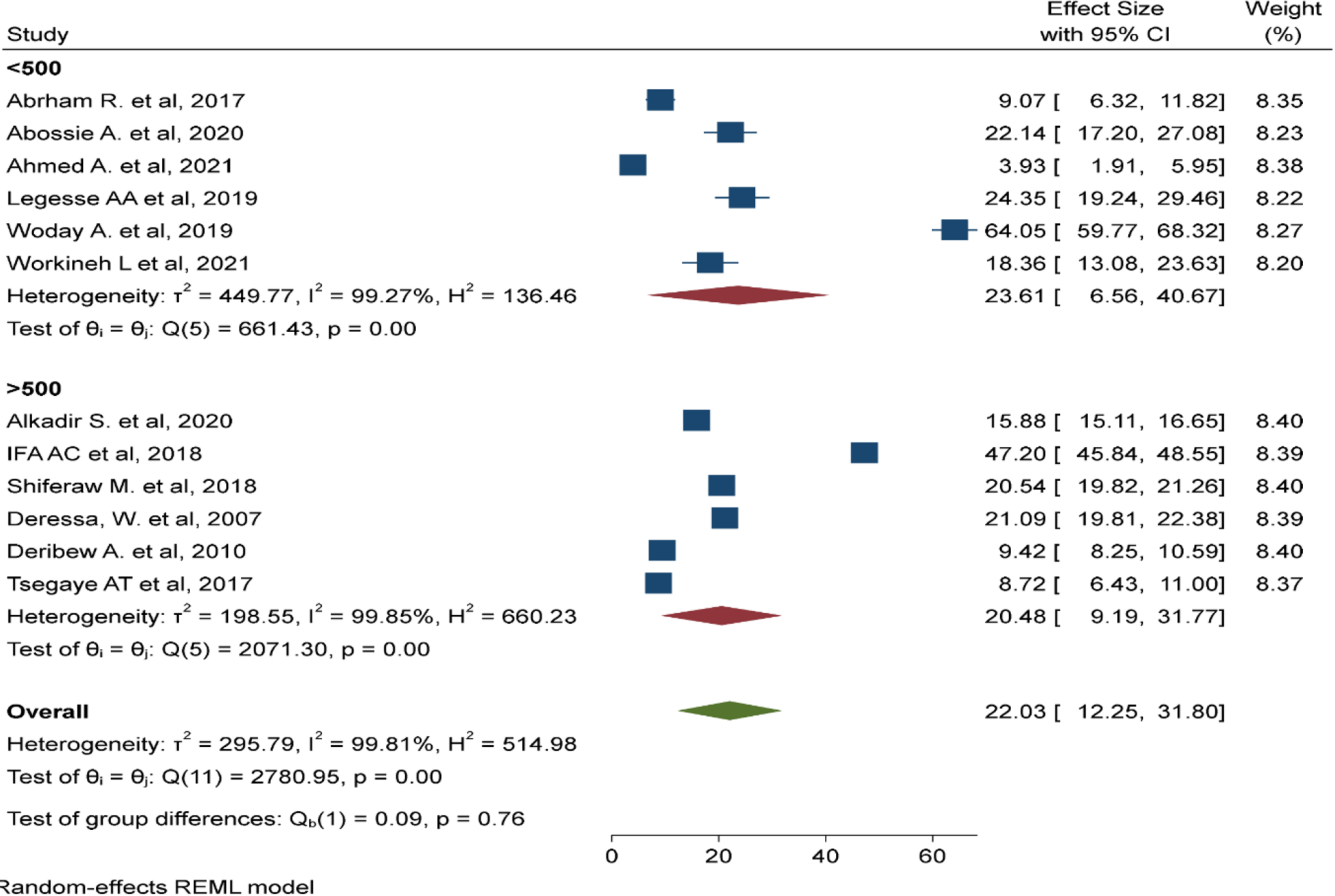
Subgroup analysis based on sample size, Ethiopia, August 2022

**Fig. 5:**
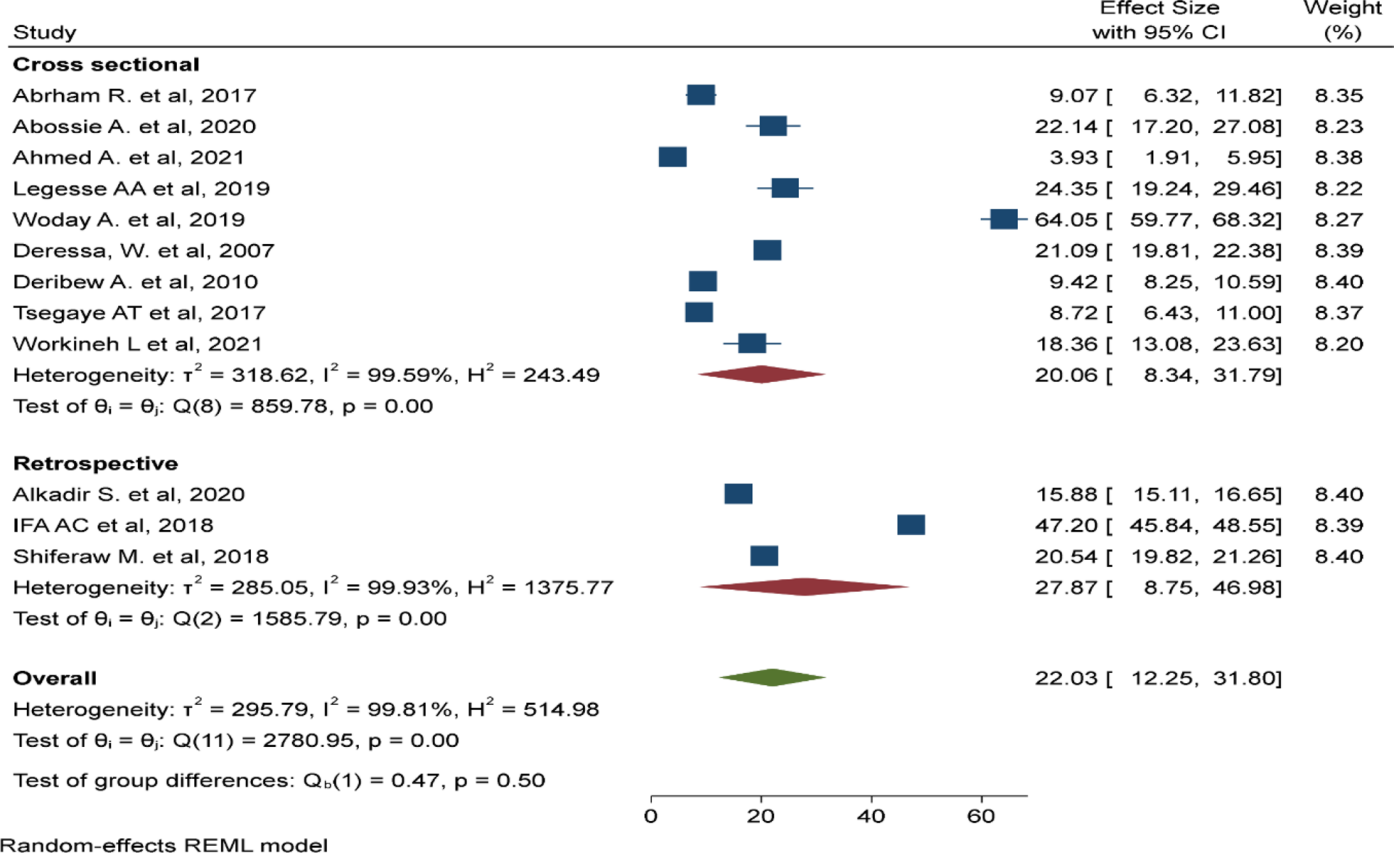
Subgroup analysis based on study design, Ethiopia, August 2022

There was a significant heterogeneity among the included studies (I^2^: 99.81, p < 0.001). As a result, sub-group analyses was done by region (Fig. 3), sample size (Fig. 4), and study design (Fig. 5). The highest pooled prevalence of under-five malaria was reported in SNNP (25.81%, 95% CI 10.22–41.39%) next to a single study in Afar region (64.5%, 95% CI 59.77–68.32%). The pooled prevalence of malaria was higher among studies with a sample size < 500 (23.61%, 95% CI 6.56–40.67%). Similarly, the pooled prevalence of malaria was higher in studies with a retrospective design (27.87%, 95% CI 8.75–46.98%).

### Publication bias and sensitivity analysis

**Fig. 6:**
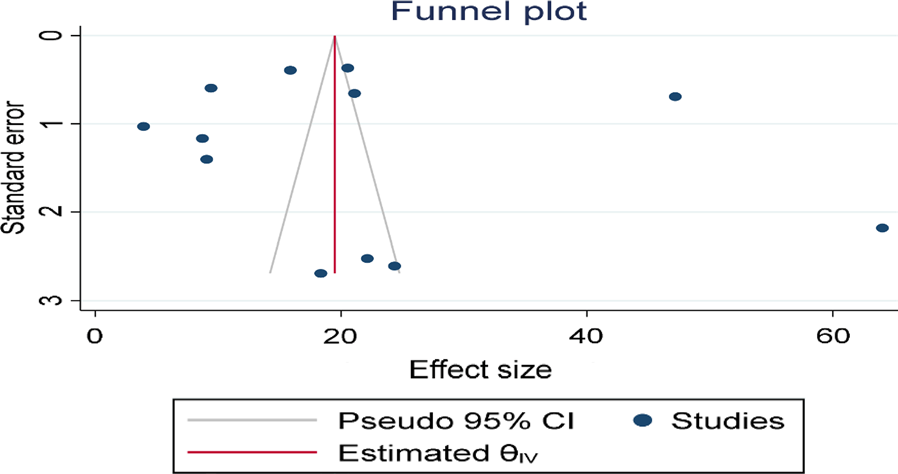
Funnel plot to determine the presence of publication bias among the included studies

**Fig. 7:**
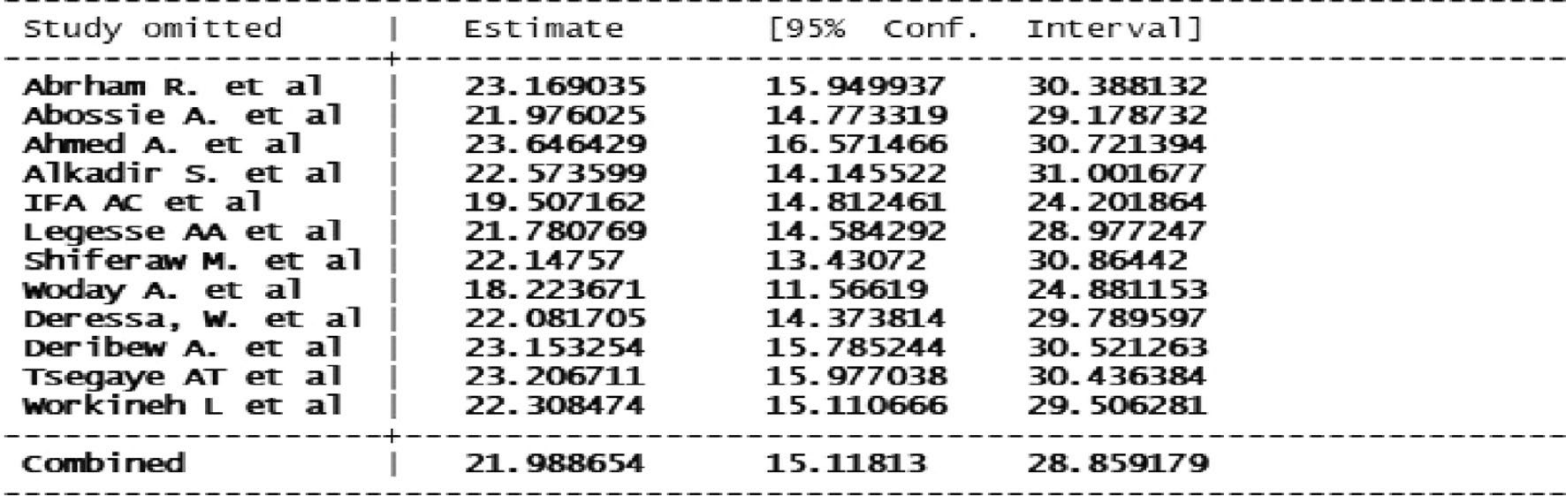
Sensitivity analysis for the study of malaria among under five children in Ethiopia, August 2022

The funnel plot showed a significant publication bias with substantial asymmetry (Fig. 6). The funnel plot is a subjective technique; as a result, objective statistical tests Egger’s test and Begg’s test [39] were done to confirm the presence of publication bias. Consequently, the Egger’s (p = 0.43) and Begg’s statistical tests (p = 0.63) showed no statistical evidence of publication bias. Sensitivity analysis was performed with the random effect model to see the effect of a single study on the overall estimate. However, the analysis showed no evidence for the influence of a single study on the overall estimate (Fig. 7).

## Risk factors of malaria in under-five children

Several primary studies were included to determine the pooled factors associated with malaria among children age under 5 years. Eleven primary studies were included to assess the association between ITN utilization, five studies for assessing the association between living near mosquito breeding sites, and five studies to determine the relationship between knowledge of malaria transmission and the prevalence of malaria among under-five children.

### Insecticide-treated mosquito net utilization

**Fig. 8:**
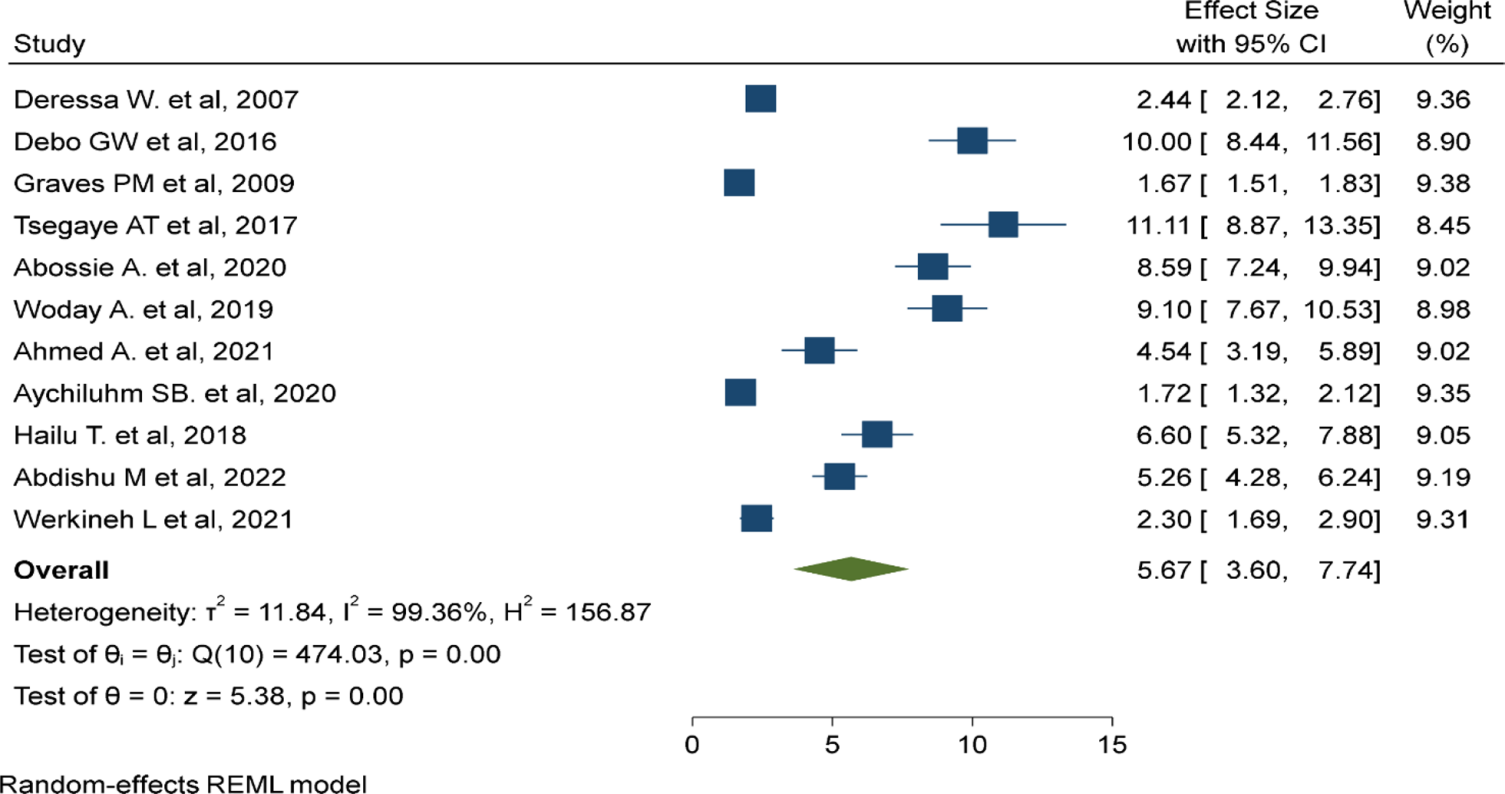
Lack of insecticide treated bed net is a risk factor of malaria among under five children

In this study, 11 primary studies were included to assess the association of ITN utilization and the prevalence of malaria among children aged under 5 years [27, 28, 30, 35, 37, 38, 40–44]. Children age under 5 years who did not utilize an ITN were nearly 6 times (AOR: 5.67, 95% CI 3.6–7.74) more likely to have malaria than children who did utilize an ITN (Fig. 8).

### Living near mosquito breeding sites

**Fig. 9:**
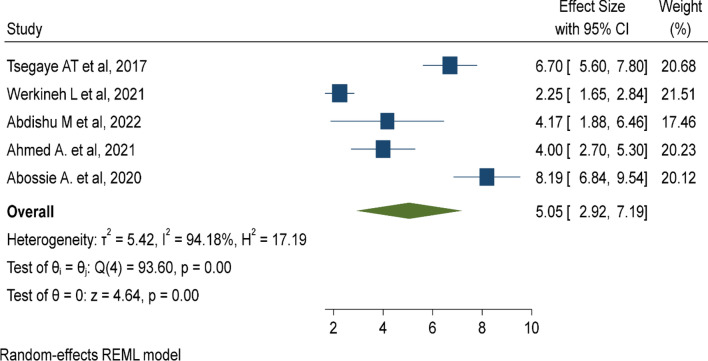
Mosquito breeding site is a risk factor of malaria among under five children

In this meta-analysis, five primary studies were included to assess the association between living near mosquito breeding sites and the rate of malaria in under-five children [28, 30, 37, 38, 44]. which was five times (AOR: 5.05, 95% CI 2.92–7.19) more likely to be infected by malaria parasites compared to children living near a non-breeding site (Fig. 9).

### Knowledge of malaria transmission

**Fig. 10:**
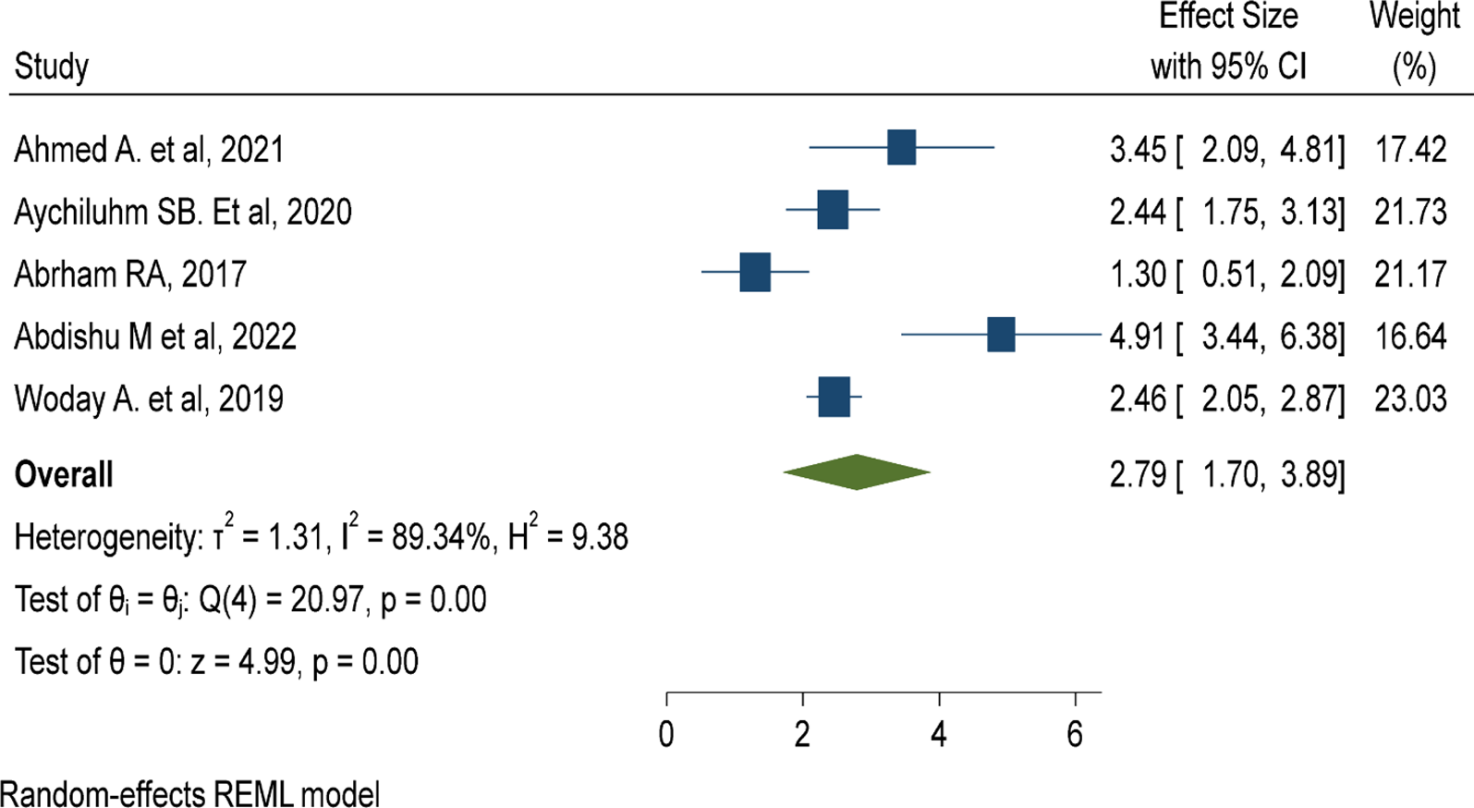
Poor knowledge of malaria transmission method is a risk factor of malaria among under five children

Five primary studies were included to determine the effect of knowledge of child caretakers about malaria transmission on the incidence of under-five malaria [27–29, 42, 44], which showed that under-five children with poor knowledge of caretakers toward malaria transmission were nearly three times (AOR: 2.79, 95% CI 1.7–3.89) at high risk of malaria compared to their counterparts (Fig. 10).

## Discussion

This study explores and synthesizes the available evidence on malaria in children under 5 years old and its associated factors in Ethiopia. By gathering, synthesizing, and pooling available studies, the finding provides strong evidence on under-five malaria in Ethiopia. The finding revealed that more than one in five children are infected with malaria *Plasmodium* species. The finding warned the 2030 national malaria elimination programme in Ethiopia [45] needs to emphasize malaria control strategies in the country.

In this study, 22.03% (95% CI 12.25%, 31.80%) under-five children were infected with malaria. The finding is comparable to the pooled estimate in sub-Saharan countries (18.8%) [46], (21%) [47], and (26%) [48], the national malaria survey in Gambella (21%) [49], a study in Uganda (19.04%) [50], the national malaria survey in Ghana (21%) [51], and the national malaria survey in Nigeria (27%) [52]. These findings pointed out that malaria is still a concern for children under 5 years old. The reason could be their low protective immunity makes them highly susceptible to malaria infection, and could be associated with poor malaria control strategies or lack of monitoring and evaluation of malaria control programmes.

The finding shows a nearly 16-fold higher estimate than the national malaria indicator survey in Ethiopia (1.4%) [49]. The Government of Ethiopia need to strengthen malaria control strategies. The finding is threefold higher than the demographic and health survey in Tanzania (7%) [53] and the malaria indicator survey in Kenya (6%) [54]. The reason could be demographic health surveys and malaria indicator surveys are conducted at national level, including low malaria-endemic areas, which thereby lower the report of under-five malaria. However, studies included in this meta-analysis were conducted in high malaria areas of Ethiopia which might increase the prevalence of under-five malaria.

Conversely, the finding was lower than the report in sub-Saharan countries (36%) [55], a study in Malawi (37%) [56], the malaria indicator survey in Liberia (45%) [57], the malaria indicator survey in south Sudan (32%) [58], and a meta-analysis in the Democratic Republic of the Congo (37.4%) [59]. The possible explanation for the discrepancy could be countries might have different malaria monitoring programmes and different levels of achievements in malaria control strategies. The reason could also be due to the fact that Ethiopia had low malaria prevalence compared to most other malaria-endemic countries in Africa [45].

Lack of access to or non-utilization of ITNs is a risk factor for higher prevalence of malaria among under-five children, which is similar to the study in Ethiopia [42], a study in Malawi [60], a study in sub-Saharan countries [47], and a study in Uganda [61]. The reason is the fact that children who utilize ITNs are not exposed to *Plasmodium* species and are less likely to get infected by the parasite. The Government should disseminate ITNs and advocate utilization to prevent high malaria transmission among under-five children.

Poor knowledge of child caretakers about malaria transmission increases the likelihood of malaria infection. The finding is similar to studies in sub-Saharan countries [62], in Rwanda [63], Ghana [64], Uganda [50], Malawi [56], and Nigeria [65]. The reason could be child caretakers who are not aware of malaria transmission are less likely to protect their child from *Plasmodium* species. Community awareness regarding malaria transmission and its prevention should be strengthened.

Residing near mosquito breeding sites such as stagnant water increases malaria transmission. The finding is similar to the pooled estimate in sub-Saharan countries [46], studies in Ethiopia [66], Malawi [67], South Africa [68], and Nigeria [65]. The reason is mosquitoes can multiply and survive in stagnant and unprotected water sources. As a result, under-five children living near these areas are at higher risk of malaria infection. Cleaning and removing mosquito breeding sites is advisable to reduce malaria in under-fives in Ethiopia.

### Strength and limitations of the study

A limitation of the study is pooling prevalence and odds ratio despite high heterogeneity. There are outliers in the included studies such as the study from Afar region this might result in an exaggerated pooled estimate. Another limitation of the study is its narrow scope, which included studies involving only children aged under 5 years. In addition, only a few factors were considered by excluding factors that were reported in a few studies this might cause bias on the factors of under-five malaria. Similarly, excluding articles that were published other than in English language might cause publication bias.

## Conclusion

The finding revealed that more than one in five children aged under 5 years were infected with malaria. The risk factors identified are mostly preventable, including ITN under-utilization, living near mosquito breeding sites, and poor knowledge of malaria transmission and its prevention methods by caretakers. The Government should strengthen malaria control strategies such as eradicating mosquito breeding sites, ensure access to ITNs for all under-five children living in malaria-endemic areas, and raise awareness regarding malaria transmission and its preventative methods.

## Supplementary Information

**Additional file 1.** PRISMA 2020 Checklist for reporting the finding.

## Data Availability

All materials and data related to this article are included in the main document of the manuscript.

## Abbreviations

EDHS: Ethiopian Demographic and Health Survey
ITN: Insecticide-treated nets
LMICs: Low and Middle-Income Countries
MeSH: Medical Subject Headings
NOS: Newcastle–Ottawa Quality Assessment Scale
PECO: Population, Exposure, Comparison, and Outcome
PRISMA: Preferred Reporting Items for Systematic Review and Meta-Analysis
SNNP: South Nation Nationality and Peoples
WHO: World Health Organization
